# Supplementary material for: Characterizing the Relationship between Expression Quantitative Trait Loci (eQTLs), DNA Methylation Quantitative Trait Loci (mQTLs), and Breast Cancer Risk Variants
Source: Cancers (Basel). 2024 May 30;16(11):2072. doi: 10.3390/cancers16112072 (PMC11171367; doi:10.3390/cancers16112072)

Supplementary Figure S1. Plot of -log10 p-values from breast cancer risk genome-wide association study (GWAS) (top), Summary-Data-Based Mendelian Randomization (SMR) analysis for associations between gene expression and breast cancer risk (middle), and SMR analysis for associations between DNA methylation and breast cancer risk (bottom). Only variants from the SMR analysis which passed the HEIDI test (pHEIDI>0.05) are shown. Dataset used - whole blood mQTL dataset, lite version of the CAGE eQTL summary data, and GWAS Summary Results: Breast Cancer Risk (2017) in Europeans.

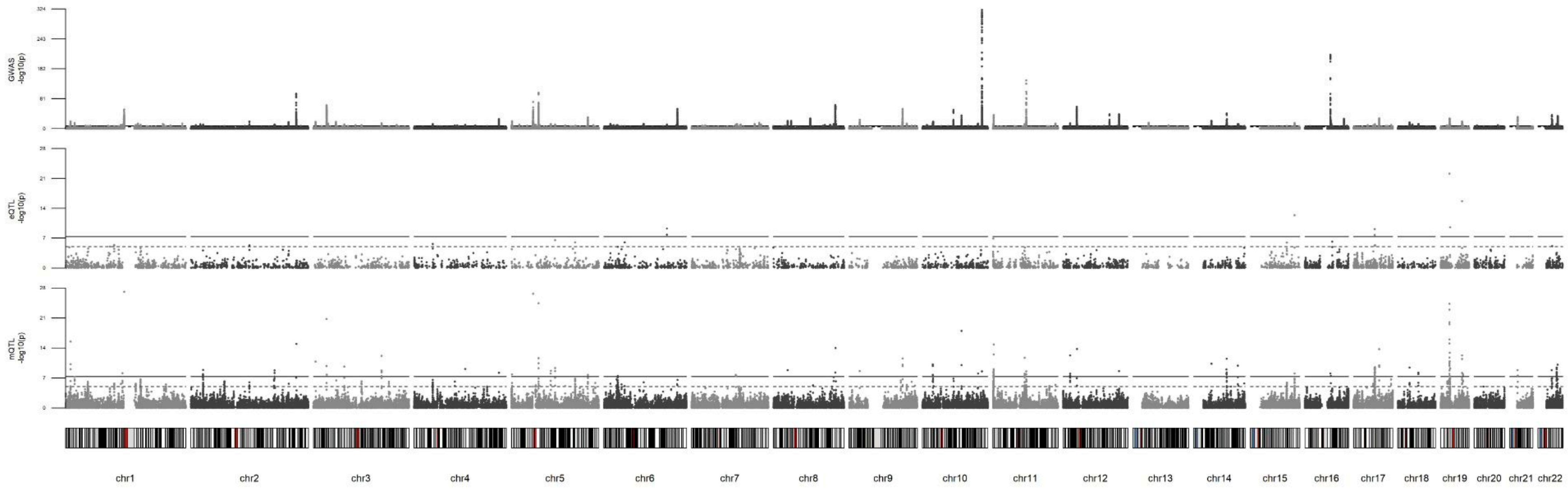

Supplementary Figure S2. Plot of -log10 p-values from breast cancer risk genome-wide association study (GWAS) (top), Summary-Data-Based Mendelian Randomization (SMR) analysis for associations between gene expression and breast cancer risk (middle), and SMR analysis for associations between DNA methylation and breast cancer risk (bottom). Only variants (on Chromosome 6) from the SMR analysis which passed the HEIDI test (pHEIDI>0.05) are shown. Dataset used - whole blood mQTL dataset, lite version of the CAGE eQTL summary data, and GWAS Summary Results: Breast Cancer Risk (2017) in Europeans.

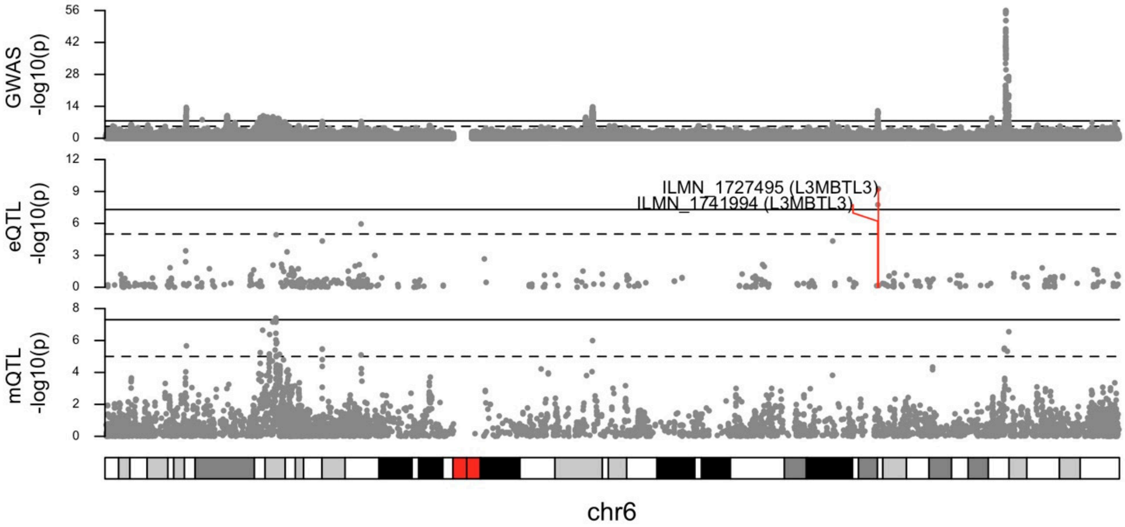

Supplementary Figure S3. Plot of -log10 p-values from breast cancer risk genome-wide association study (GWAS) (top), Summary-Data-Based Mendelian Randomization (SMR) analysis for associations between gene expression and breast cancer risk (middle), and SMR analysis for associations between DNA methylation and breast cancer risk (bottom). Only variants (on Chromosome 15) from the SMR analysis which passed the HEIDI test (pHEIDI>0.05) are shown. Dataset used - whole blood mQTL dataset, lite version of the CAGE eQTL summary data, and GWAS Summary Results: Breast Cancer Risk (2017) in Europeans.

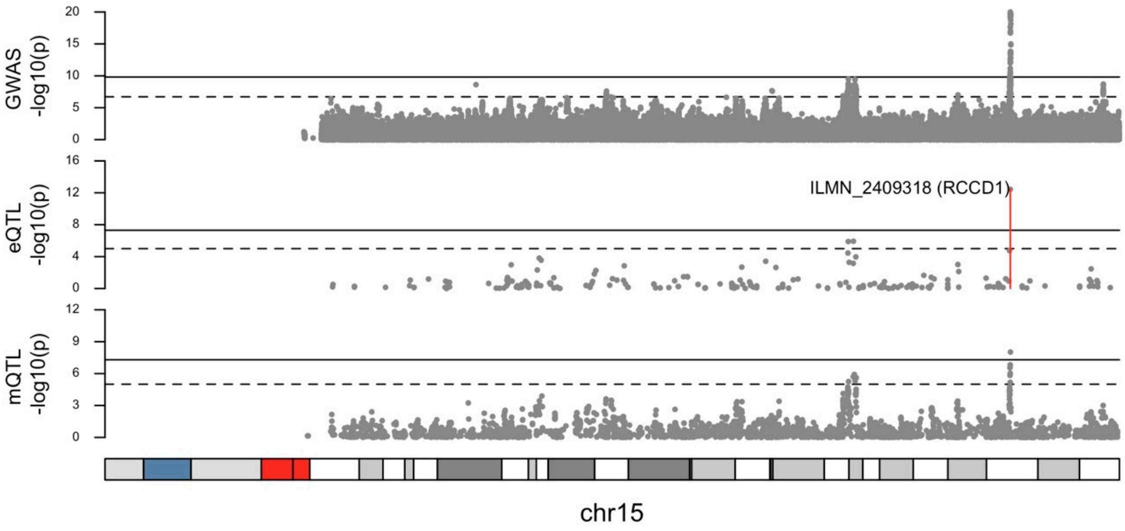

Supplementary Figure S4. Plot of -log10 p-values from breast cancer risk genome-wide association study (GWAS) (top), Summary-Data-Based Mendelian Randomization (SMR) analysis for associations between gene expression and breast cancer risk (middle), and SMR analysis for associations between DNA methylation and breast cancer risk (bottom). Only variants (on Chromosome 17) from the SMR analysis which passed the HEIDI test (pHEIDI>0.05) are shown. Dataset used - whole blood mQTL dataset, lite version of the CAGE eQTL summary data, and GWAS Summary Results: Breast Cancer Risk (2017) in Europeans.

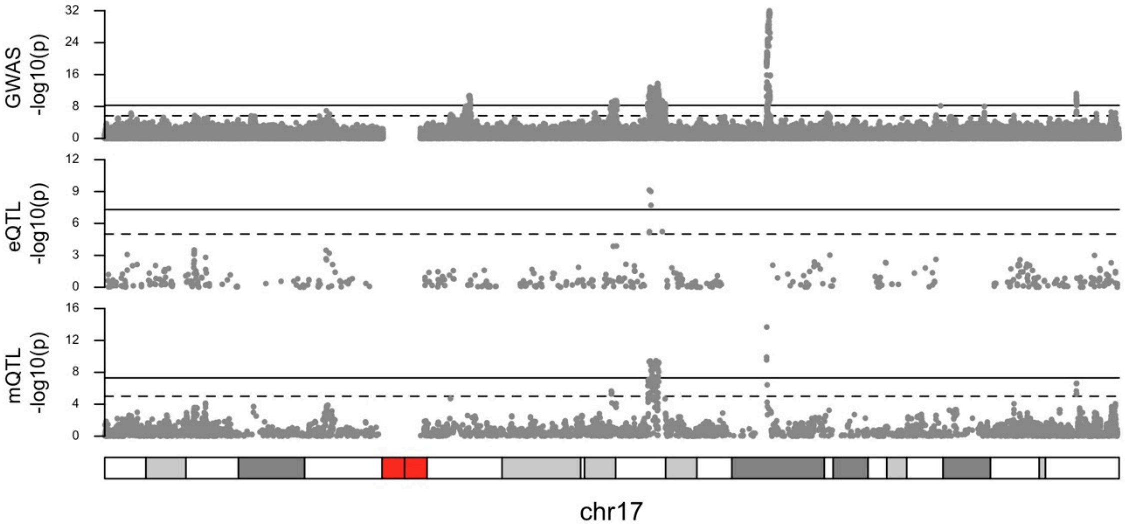

Supplementary Figure S5. Using the eQTLGen Consortium data, the plot of -log10 p-values from breast cancer risk genome-wide association study (GWAS) (top), Summary-Data-Based Mendelian Randomization (SMR) analysis for associations between gene expression and breast cancer risk (middle), and SMR analysis for associations between DNA methylation and breast cancer risk (bottom). Only variants from the SMR analysis which passed the HEIDI test (pHEIDI>0.05) are shown. Dataset used - whole blood mQTL dataset, QTLGen Consortium data (eQTL), and GWAS Summary Results: Breast Cancer Risk (2017) in Europeans.

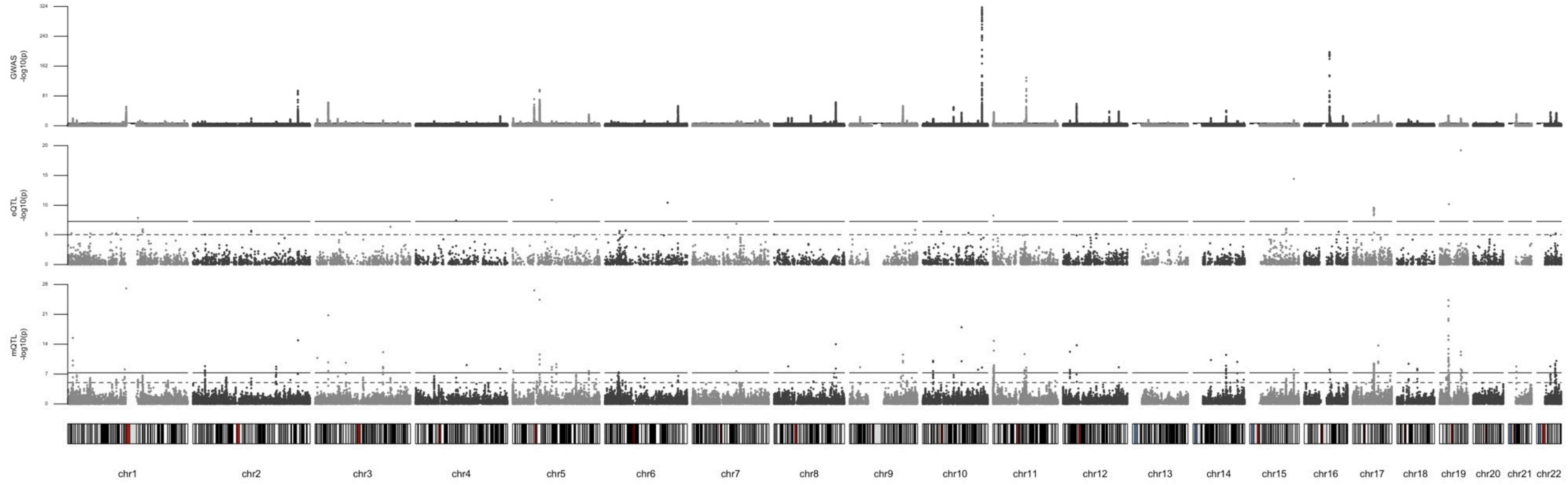

Supplementary Figure S6. Plot of -log10 p-values from breast cancer risk genome-wide association study (GWAS) (top), Summary-Data-Based Mendelian Randomization (SMR) analysis for associations between gene expression and breast cancer risk (middle), and SMR analysis for associations between DNA methylation and breast cancer risk (bottom). Only variants (on Chromosome 1) from the SMR analysis which passed the HEIDI test (pHEIDI>0.05) are shown. Dataset used - whole blood mQTL dataset, QTLGen Consortium data, and GWAS Summary Results: Breast Cancer Risk (2017) in Europeans.

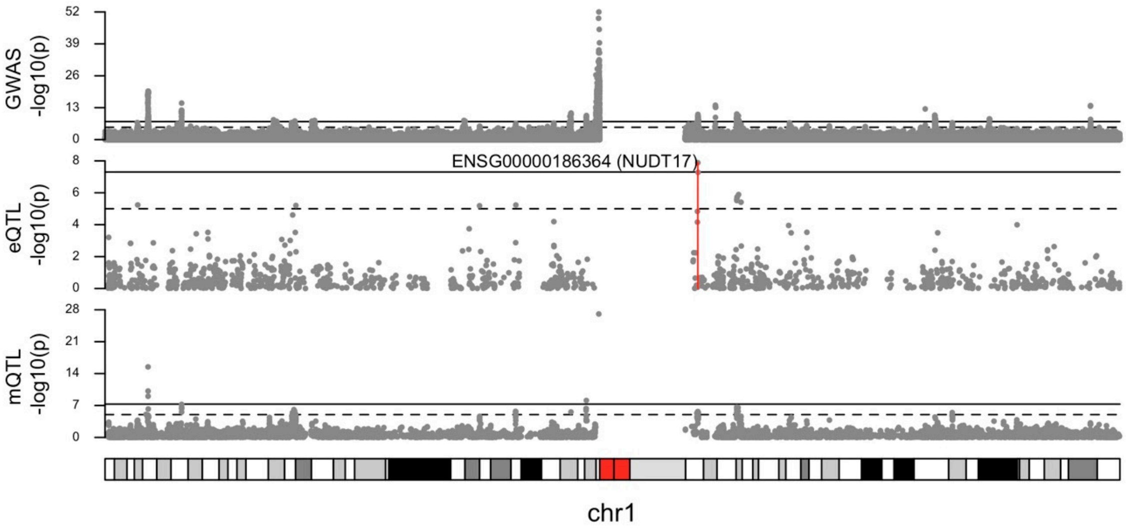

Supplementary Figure S7. Plot of -log10 p-values from breast cancer risk genome-wide association study (GWAS) (top), Summary-Data-Based Mendelian Randomization (SMR) analysis for associations between gene expression and breast cancer risk (middle), and SMR analysis for associations between DNA methylation and breast cancer risk (bottom). Only variants (on Chromosome 4) from the SMR analysis which passed the HEIDI test (pHEIDI>0.05) are shown. Dataset used - whole blood mQTL dataset, QTLGen Consortium data, and GWAS Summary Results: Breast Cancer Risk (2017) in Europeans.

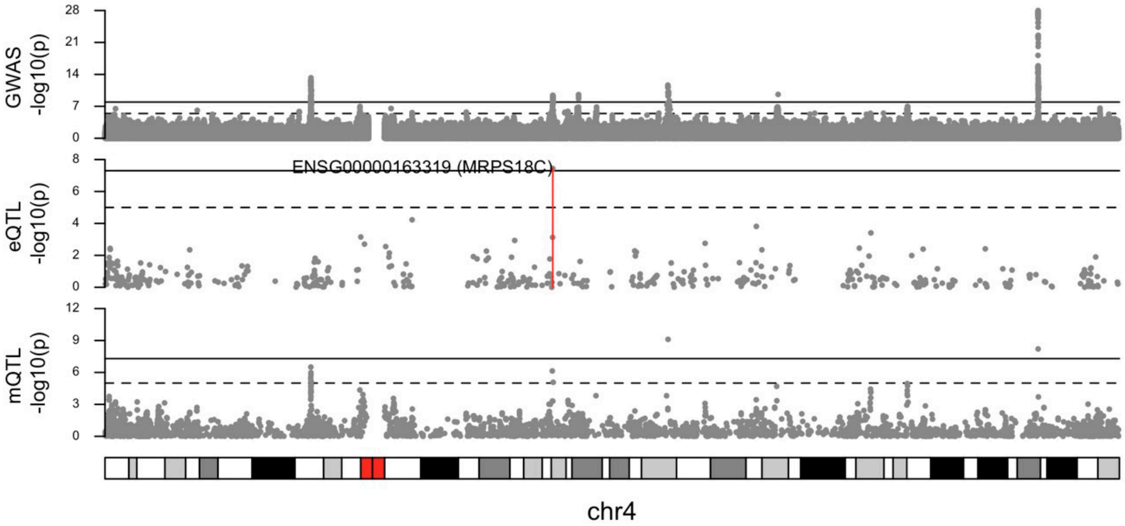

Supplementary Figure S8. Plot of -log10 p-values from breast cancer risk genome-wide association study (GWAS) (top), Summary-Data-Based Mendelian Randomization (SMR) analysis for associations between gene expression and breast cancer risk (middle), and SMR analysis for associations between DNA methylation and breast cancer risk (bottom). Only variants (on Chromosome 5) from the SMR analysis which passed the HEIDI test (pHEIDI>0.05) are shown. Dataset used - whole blood mQTL dataset, QTLGen Consortium data, and GWAS Summary Results: Breast Cancer Risk (2017) in Europeans.

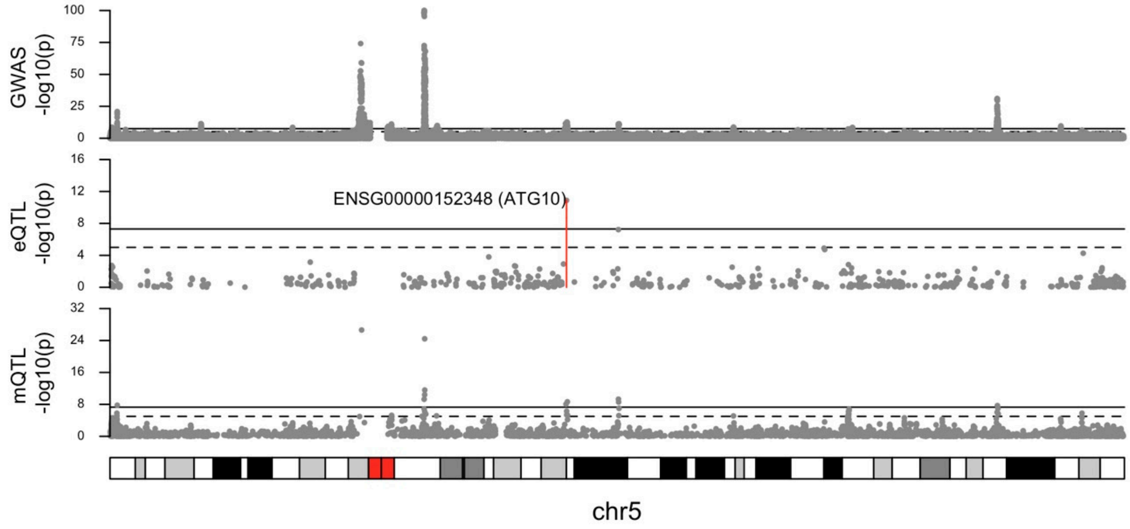

Supplementary Figure S9. Plot of -log10 p-values from breast cancer risk genome-wide association study (GWAS) (top), Summary-Data-Based Mendelian Randomization (SMR) analysis for associations between gene expression and breast cancer risk (middle), and SMR analysis for associations between DNA methylation and breast cancer risk (bottom). Only variants (on Chromosome 6) from the SMR analysis which passed the HEIDI test (pHEIDI>0.05) are shown. Dataset used - whole blood mQTL dataset, QTLGen Consortium data, and GWAS Summary Results: Breast Cancer Risk (2017) in Europeans.

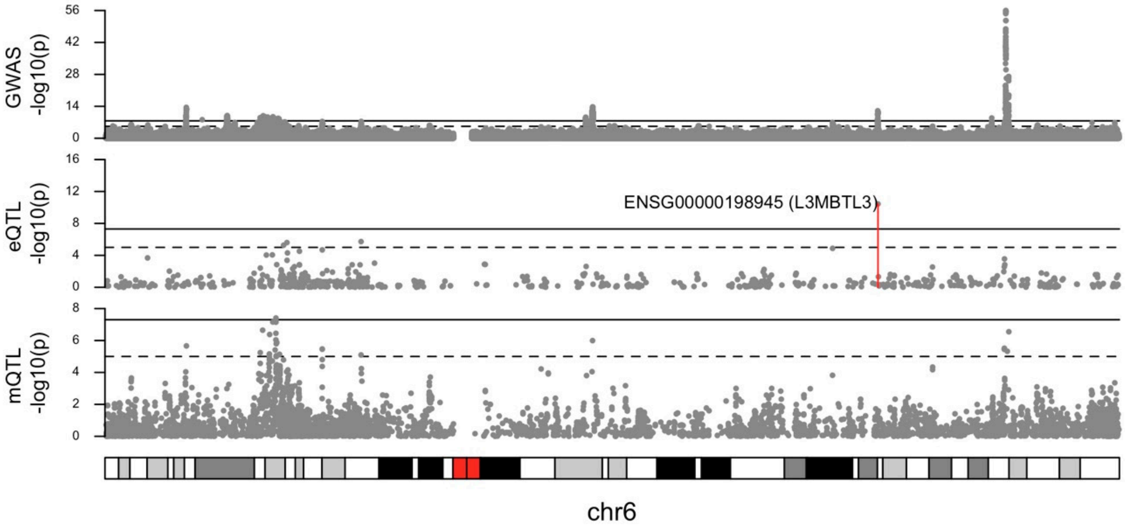

Supplementary Figure S10. Plot of -log10 p-values from breast cancer risk genome-wide association study (GWAS) (top), Summary-Data-Based Mendelian Randomization (SMR) analysis for associations between gene expression and breast cancer risk (middle), and SMR analysis for associations between DNA methylation and breast cancer risk (bottom). Only variants (on Chromosome 11) from the SMR analysis which passed the HEIDI test (pHEIDI>0.05) are shown. Dataset used - whole blood mQTL dataset, QTLGen Consortium data, and GWAS Summary Results: Breast Cancer Risk (2017) in Europeans.

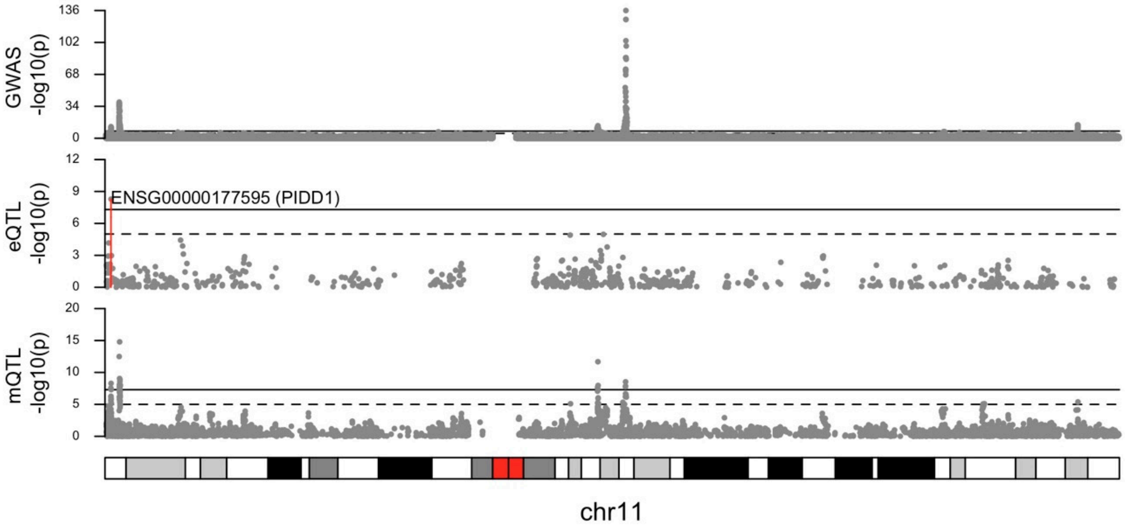

Supplementary Figure S11. Plot of -log10 p-values from breast cancer risk genome-wide association study (GWAS) (top), Summary-Data-Based Mendelian Randomization (SMR) analysis for associations between gene expression and breast cancer risk (middle), and SMR analysis for associations between DNA methylation and breast cancer risk (bottom). Only variants (on Chromosome 15) from the SMR analysis which passed the HEIDI test (pHEIDI>0.05) are shown. Dataset used - whole blood mQTL dataset, QTLGen Consortium data, and GWAS Summary Results: Breast Cancer Risk (2017) in Europeans.

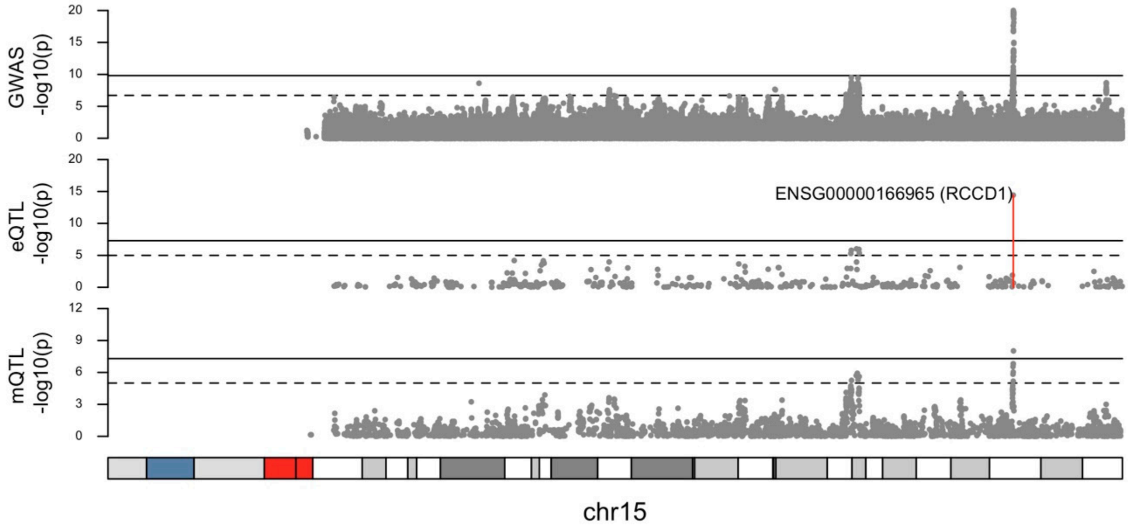

Supplementary Figure S12. Plot of -log10 p-values from breast cancer risk genome-wide association study (GWAS) (top), Summary-Data-Based Mendelian Randomization (SMR) analysis for associations between gene expression and breast cancer risk (middle), and SMR analysis for associations between DNA methylation and breast cancer risk (bottom). Only variants (on Chromosome 17) from the SMR analysis which passed the HEIDI test (pHEIDI>0.05) are shown. Dataset used - whole blood mQTL dataset, QTLGen Consortium data, and GWAS Summary Results: Breast Cancer Risk (2017) in Europe.

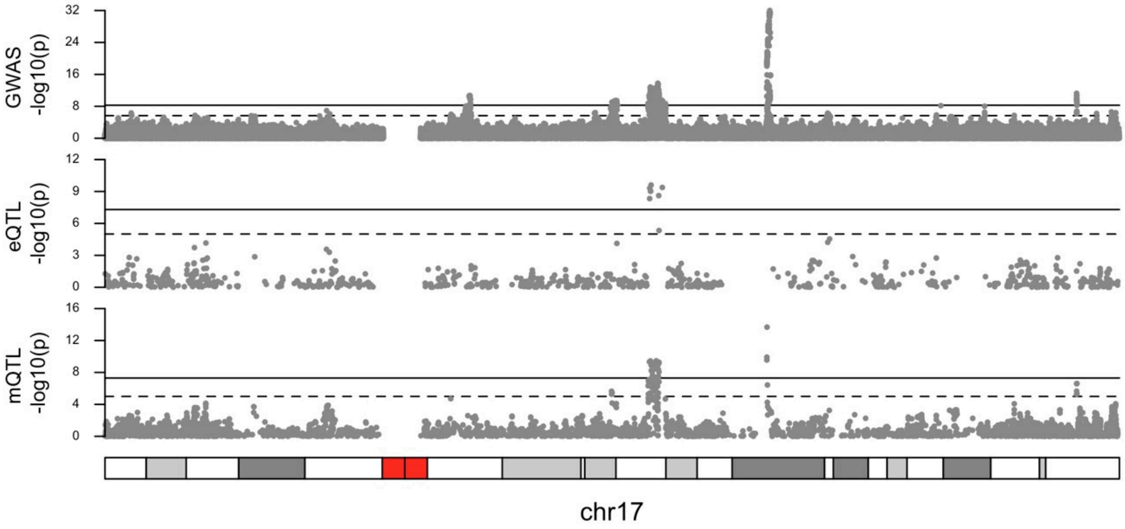

Supplementary Figure S13. Plot of -log10 p-values from breast cancer risk genome-wide association study (GWAS) (top), Summary-Data-Based Mendelian Randomization (SMR) analysis for associations between gene expression and breast cancer risk (middle), and SMR analysis for associations between DNA methylation and breast cancer risk (bottom). Only variants (on Chromosome 19) from the SMR analysis which passed the HEIDI test (pHEIDI>0.05) are shown. Dataset used - whole blood mQTL dataset, QTLGen Consortium data, and GWAS Summary Results: Breast Cancer Risk (2017) in Europe.

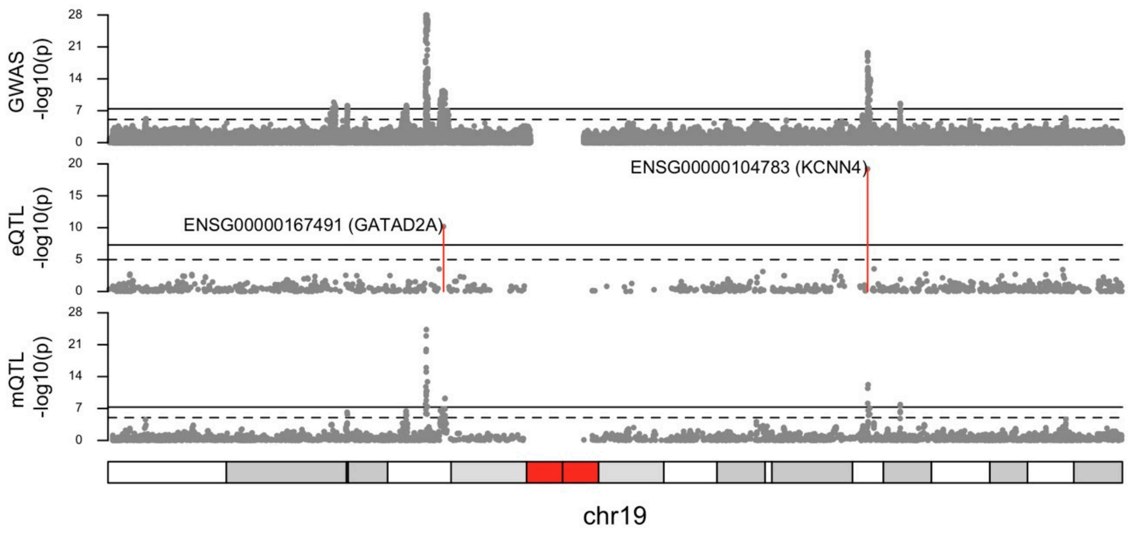

Supplementary Figure S14. Overlap in high risk individuals in 1) breast cancer cases and 2) controls, identified by GWAS PRS weighted by Michailidou et al. (white) and Mavaddat et al. (red). High risk was based on the threshold at the 80th centile of the control group for each score.

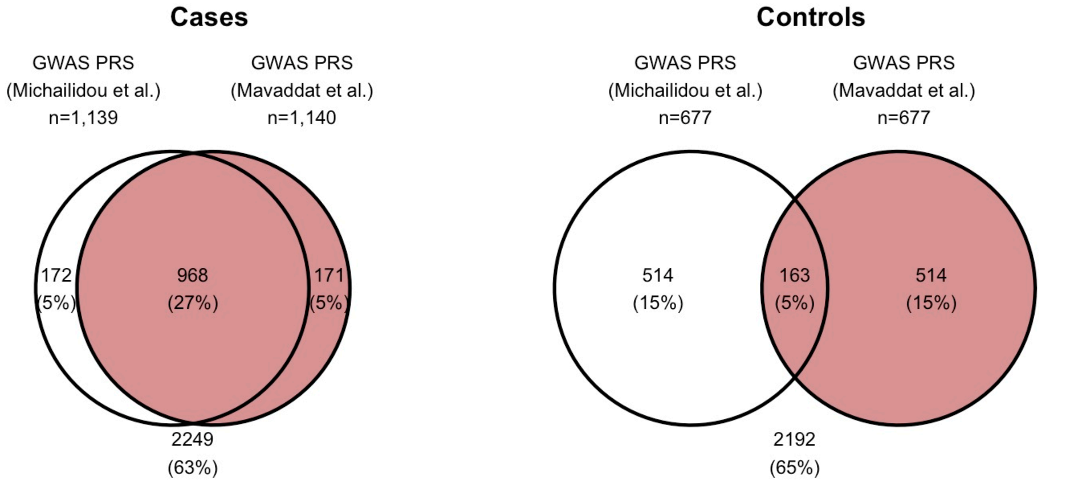

Supplementary Figure S15. Flowchart for individuals selected in (A) breast cancer patients from Singapore Breast Cancer Cohort (SGBCC), and (B) ethnicity- and age-matched controls from the Singapore Multi-Ethnic Cohort (MEC) study.

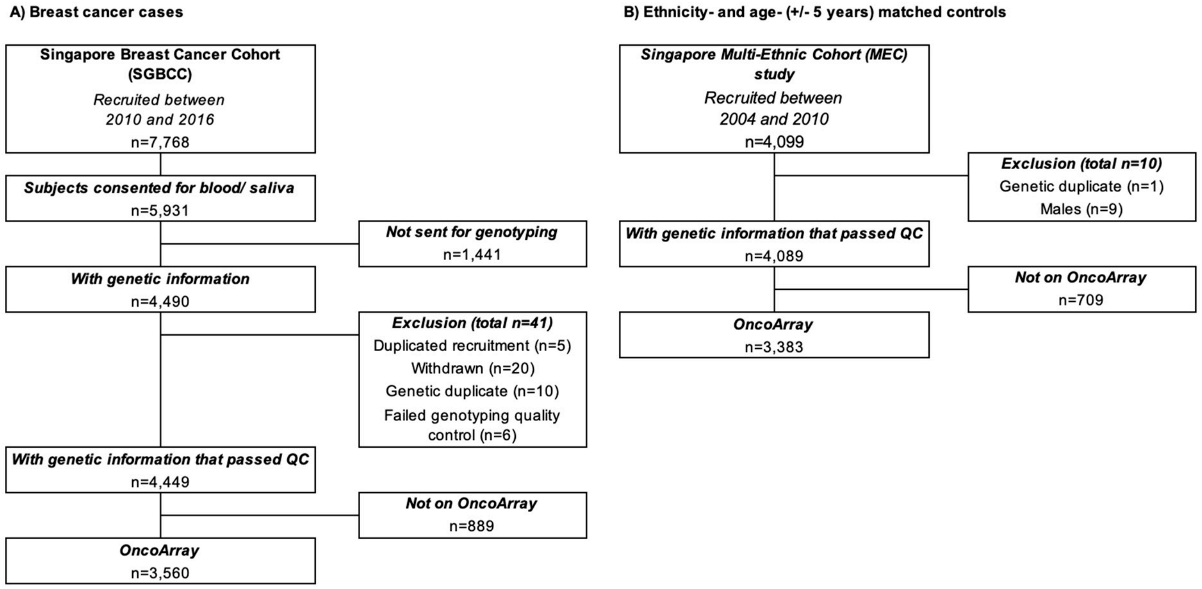

Supplement: Supplementary file 1 [file cancers-16-02072-s001.zip › Supplementary figures.pdf]
